# Development and validation of the job stressor scale for specialty nurses

**DOI:** 10.3389/fpsyg.2024.1450334

**Published:** 2024-09-09

**Authors:** Panpan Zhang, Wenqiong Lin, Songyao Li, Yaru Li, Jili Wei, Huiyi Zhang, Bo Zhang, Ziru Fang, Rui Guo, Hongmei Zhang

**Affiliations:** ^1^Nursing Department, Henan Provincial People’s Hospital, Zhengzhou, China; ^2^Nursing Department, People’s Hospital of Zhengzhou University, Zhengzhou, China; ^3^Nursing Department, People’s Hospital of Henan University, Zhengzhou, China; ^4^Henan Provincial Key Medicine Laboratory of Nursing, Zhengzhou, China; ^5^Zhengzhou University People’s Hospital, Zhengzhou, China; ^6^Department of Medical Oncology, First Affiliated Hospital of Zhengzhou University, Zhengzhou, China; ^7^Anesthesia Intensive Care Unit, Henan Provincial People’s Hospital, Zhengzhou, China; ^8^Department of Pediatrics, Henan Provincial People’s Hospital, Zhengzhou, China

**Keywords:** specialties, nurses, stressor, scale, development, evaluation

## Abstract

**Objectives:**

Specialty nurses play a crucial role in specialized nursing practice, teaching, management, and research. These nurses often face significant work pressure; therefore, scientifically and effectively assessing their job stress and its sources is vital for enhancing the quality of their work. However, there is currently a dearth of verified assessment tools for measuring job stressors among specialty nurses. Therefore, this study aimed to develop and test an instrument to assess the job stressors applicable to specialty nurses.

**Methods:**

We conducted a multiphase mixed-methods study. The initial scale items were developed from a literature review and structured interviews. The scale was then refined through two rounds of expert consultation (*N* = 14) and a primary test (*N* = 20). A main survey (*N* = 552) was then conducted to evaluate the scale’s construct validity and reliability using confirmatory factor analysis (CFA) and exploratory factor analysis (EFA).

**Results:**

The final scale comprises four dimensions with 27 items. The factors included “specialized nursing and work,” “workload and time allocation,” “patient care,” and “work resources and environment.” The EFA explained 69.10% of the variance, while the CFA confirmed a good model fit. The content validity index was 0.980 at the scale level and 0.790–1.000 at the item level. The scale’s reliability was supported by its high Cronbach’s *α* (0.958), test–retest reliability (0.946), and split-half reliability (0.868).

**Conclusion:**

Our findings indicate that the job stressor scale developed in this study is valid and reliable, and is recommended for use among specialty nurses to assess their stressors.

## 1 Introduction

Specialty nurses are registered nurses who have worked in a certain specialized field, undergone systematic professional training in the theory and practice of that field, or obtained corresponding qualifications; these nurses are able to skillfully apply their nursing knowledge—along with current technology—to provide specialized services for nursing service recipients (Nie et al., 2022). Specialty nurses can effectively manage their patients’ condition, promote their recovery, shorten their hospital stay, and improve their quality of life. These nurses represent an important human resource (Whitehead et al., 2019; Qin et al., 2022a,b). The United States initiated training of specialty nurses as early as 1956 (Mayo et al., 2017). Since the 1980s, developed countries such as Canada, the United Kingdom, Australia, and Japan have successively trained a large number of specialty nurses, with more than 200 specialized nursing fields established after more than a century of evaluation (Wang et al., 2016). The development of specialty nurses in China started relatively late, in comparison; however, after over 20 years of exploration, it has reached a certain scale. Specialty nurses’ training in China now covers 33 specialty fields, with the number of specialty nurses reaching 53,000—a growth rate of approximately 53% over 5 years (Ding et al., 2020; Wu, 2022). The team of specialty nurses worldwide is clearly expanding continuously.

With this continuous growth, enabling this workforce to fully utilize their roles via appropriate application and management strategies is a key concern for hospital management. Owing to the late development of specialty nurses in China, current research mainly focuses on their training and assessment. Therefore, utilization and management systems for specialty nurses remain unclear (He et al., 2021). This has led to most specialty nurses needing to undertake a lot of specialized nursing, management, research, and teaching work, in addition to their general clinical nursing duties; this, in turn, leads to a relatively high level of work pressure in the field (Ding et al., 2021; Ma and Li, 2022). In developed countries, such as the United States, the training, certification, and application systems of specialty nurses are relatively well established; moreover, specialty nurses not only have independent prescribing authority, they are also qualified to perform advanced medical practices, such as running specialized clinics and performing surgeries (Unsworth et al., 2024). However, this entails higher educational requirements, a more competitive environment, and subsequent increases in responsibility and pressure. Job stress is therefore a common issue faced by specialty nurses worldwide.

“Job stressors” encompass a variety of stimuli present in the work environment or among employees, while “job stress” refers to the psychological and physiological tension that arises when individuals experience these stimuli (Zhang, 2018). For specialty nurses, work stress arises from the cumulative effects of stressors encountered in nursing practice (Chen et al., 2020). Prolonged exposure to stressors, such as heavy workloads, management issues, and professional conflicts, precipitates ongoing psychological and behavioral responses in specialty nurses, which, if not addressed adequately, may not only harm the physical and mental health of the individual but also lead to job burnout, ultimately impacting work quality (Shah et al., 2021; Zhang et al., 2022). Consequently, it is imperative to develop targeted assessment tools based on an understanding of the job stressors faced by specialty nurses, providing a basis for the formulation of intervention strategies to alleviate work stress among them.

Currently, there exists both universal and specific tools for assessing nurses’ stressors—domestically and internationally; among these, specific assessment tools mainly involve the fields of emergency medicine (Yuwanich et al., 2018), psychiatry (Yada et al., 2011), ICU nursing (Bailey et al., 1980), and pediatric oncology (Hinds et al., 1990). As these are limited to their applicable populations, they have not been widely used (Tian et al., 2022). Scholars in related fields mostly use universal assessment tools to assess the level of stress in nurses, such as the Perceived Stress Scale (PSS; Cohen et al., 1983), the Nursing Stress Scale (NSS; Gray-Toft and Anderson, 1981), and Chinese Nurses’ Work Stressors Scale (Li and Liu, 2000).

The PSS, proposed and developed by Cohen et al. (1983), is a widely used stress assessment scale that measures individuals’ stress perceptions within their situation over a specific period, such as the past month. However, although the PSS can measure the perceived stress level of all population groups over a period, it may not be able to accurately capture the specific pressures experienced by nurses. Additionally, with only 10 items, it may not adequately measure the perceived stress. The NSS, developed by Gray-Toft and Anderson (1981), has been translated into multiple languages and is a classic tool for assessing nurses’ stress. However, over the last 40 years since its development, many changes have occurred in the nursing field, and the pressures faced by nurses have also changed. Some of the NSS’ content may lack specificity and may require updating. Furthermore, PSS and NSS lack standardization and are not constructed based on a deep understanding of the stressors specific to specialty nurses (Tian et al., 2022). This makes it difficult for managers to accurately identify the sources of work stress for specialty nurses and develop targeted interventions.

To address this gap, Li and Liu (2000) developed the Chinese Nurse Stressor Scale, specifically tailored to reflect the realities faced by Chinese nurses, drawing on the NSS framework. The scale is based on the actual status of Chinese nurses and, combined with the NSS, is widely used to measure nurses’ stress (Zhu et al., 2020). However, this scale is only suitable for Chinese nurse populations, and may not fully capture the stressors related to specialty nurses. Due to differences in role positioning, job responsibilities, and work requirements, the sources of work stress for specialty nurses differ from those for other clinical nurses. Currently, there is a scarcity of job stressor assessment tools tailored to the characteristics of specialty nurses (Qin et al., 2022a,b).

Robbins (1997) proposed their stress model to study work-related stress within organizations. This model can be used to explain and predict the psychological and behavioral responses of individuals when facing stressful situations (Robbins, 1997). The Robbins stress model provides a comprehensive framework that would be appropriate for understanding the sources of work stress among specialty nurses. By identifying and analyzing stressors from various perspectives—such as environmental, organizational, and individual factors—we can better understand the origins and nature of work stress among specialty nurses (Robbins, 1997; Wei et al., 2023).

Therefore, given the limited research on work stress among specialty nurses and the need for a comprehensive understanding of its sources and levels. Guided by the Robbins stress model, this study developed a job stressor scale for specialty nurses, based on domestic and foreign scales such as the NSS.

## 2 Methods

### 2.1 Study design

We conducted a multiphase mixed-methods study, divided into three phases: Phase 1 involved a comprehensive literature review and structured interviews to explore and establish the item pool. Phase 2 involved utilizing the Delphi method to establish an initial draft of the scale items. During this phase, a primary test was conducted among specialist nurses to refine the phrasing of the scale items, thus preparing the first draft. Phase 3 encompassed main surveys to modify the content and structure of the scale using factor analysis and to evaluate the scale’s validity and reliability to finalize its development.

### 2.2 Theoretical framework

Our study was guided by Robbins’ theoretical stress model, which categorizes potential stress sources into environmental, organizational, and personal factors (Robbins, 1997). Environmental factors, such as macro-level stress sources, have potential effects on specialty nurses, while organizational and personal factors have a more direct impact on their stress. Our study used this model as a theoretical framework to formulate a structured interview outline and compile scale items.

### 2.3 Procedure

#### 2.3.1 Phase 1: generation of the item pool

##### 2.3.1.1 Literature research method

The literature review methods comprised two main parts: the first aimed to determine the theoretical structure of the job stressor scale for specialty nurses, while the second was to determine the specific content of the job stressor scale for specialty nurses.

The data sources included the following electronic databases: CNKI, VIP, Wanfang, PubMed, and Web of Science. The search terms “stress,” “work stress,” “stressors,” and “scale” were utilized in a combination of subject headings and free-text queries to retrieve studies on work stress among specialty nurses. The search covered all records available from the inception of each database to January 31, 2023.

##### 2.3.1.2 Structured interviews

To clarify the framework of the theoretical dimensions of job stressors, as faced by specialty nurses, we conducted structured interviews to gain a deeper understanding of the essence and implications of these stressors. We employed convenience sampling to select specialty nurses from a tertiary hospital in Henan Province as interview participants, with the inclusion criteria being (1) specialty nurses with provincial or higher qualifications and (2) possessing at least 1 year of experience in specialized nursing practice. All interviewees consented to having the entire process recorded. Structured, in-depth interviews were conducted between March and April 2023. Examples of guiding questions were as follows: (1) What are your perceptions of specialty nursing work? (2) What are the unique characteristics of the work undertaken by specialty nurses? (3) How do you feel about your current work schedule? (4) How do you evaluate the work environment and resource allocation of specialty nurses? (5) How do you perceive the support provided by your leaders, colleagues, and family members? (6) What situations in your work make you feel stressed? (7) What measures would you suggest to alleviate stress among specialty nurses?

Before each interview, we explained the purpose, methods, and content of the interview to the interviewees, ensuring strict confidentiality. The interviews were conducted face-to-face in a quiet office setting, with each session carefully timed to last 20–30 min. We followed an interview guide; recorded the interviews; listened carefully without guiding or implying; and observed the interviewees’ non-verbal expressions such as tone, intonation, and body language. We also asked follow-up questions when appropriate, to obtain valuable information. Audio recordings were transcribed into text within 24 h following the interviews. According to the principle of data saturation in qualitative research as the criterion for sample size, interviews should be terminated when no new themes emerge (Englander, 2016). After completing insightful and comprehensive interviews with 16 specialty nurses across 12 professional fields—including intensive care, operating rooms, maternal and child care, and pediatrics—no new themes emerged. The data were analyzed using Colaizzi’s seven-step method (Colaizzi, 1978).

#### 2.3.2 Phase 2: expert inquiry and item improvement

##### 2.3.2.1 Expert inquiry

The Delphi method is a “back-to-back” survey method in which experts evaluate the importance and applicability of various content and dimensions (Hasson et al., 2000). We invited 14 experts from different institutions for consultations between May and August, 2023. The inclusion criteria were as follows: (1) having been engaged in clinical nursing, nursing management, psychological nursing, or teaching for 15 years or more; (2) having a bachelor’s degree or higher; and (3) holding an intermediate or higher professional title. The reliability analysis indicators for the experts mainly included (1) expert positive coefficient, reflecting the effective recovery rate of the consultation questionnaires; (2) expert coordination coefficient, measured by the chi-squared test results of Kendall’s harmony coefficient, ranging from 0 to 1 with higher values indicating greater expert coordination; and (3) expert authority degree, calculated using the formula: (expert judgment coefficient + expert familiarity coefficient)/2, with higher scores indicating greater authority.

The consultation questionnaire included an introduction, a basic information questionnaire section, a table for assessing the correlation and importance of items, and a questionnaire regarding familiarity and judgment. Upon securing the experts’ consent to participate via telephone, email, or WeChat, the consultation questionnaire was dispatched with a request to complete it within a two-week period. The experts self-assessed their familiarity and judgment, based on these indicators. A five-point Likert scale, ranging from 1 = “very unimportant” to 5 = “very important,” was employed to evaluate the importance of each item. Similarly, a 5-point scale, ranging from 1 = “highly irrelevant” to 5 = “highly relevant,” was used to assess the correlation between items and their respective dimensions. Taking an average importance score of ≥3.5 and a coefficient of variation of ≤0.25 as the criteria for item selection, combined with expert opinions, we deleted, modified, or supplemented items to shape the initial draft of the job stressor scale for specialty nurses.

##### 2.3.2.2 Primary test

This stage was aimed at confirming the accuracy of the scale’s item descriptions. A primary test involving 20 specialty nurses was conducted in September 2023, to gather feedback on the scale. This feedback led to adjustments in the descriptions of certain items. The final version of the job stressor scale for specialty nurses was then formulated. The participants completed the primary test within 3–5 min.

#### 2.3.3 Phase 3: main survey

##### 2.3.3.1 Participant and sample size

Convenience sampling was employed to select specialty nurses as participants from two tertiary general hospitals in Zhengzhou, Henan Province, between October and November, 2023. The inclusion criteria were identical to those used for the structured interviews. Sample size estimation: to test the validity of our scale, an EFA was used. The required sample size was shown to be 5–10 times the number of items (Li, 2018). The sample size calculated for the CFA was therefore >200 (Wu, 2010a,b). The test version of the scale consisted of 27 items. Considering 20% invalid questionnaires, the sample size was estimated to have to be 362–524 specialty nurses. This study received approval from the Ethics Committee of Zhengzhou University, Henan, China (ZZUIRB2023192).

##### 2.3.3.2 Questionnaires

The questionnaire included participants’ demographic data and the test version of the job stressor scale for specialty nurses. The demographic data included participants’ age, gender, educational background, professional title, department, and years of experience. The test version of the job stressor scale for specialty nurses comprises items that are rated using a five-point Likert scale, ranging from 1 = “I do not experience this feeling” to 5 = “I experience this feeling very strongly.” As the score increased, there was greater alignment of the described situation with the stressor experienced by the participant.

### 2.4 Data collection

We converted the questionnaire to an electronic version using the *Questionnaire Star* platform. After receiving approval from the nursing department director at the target hospital, the nursing manager distributed the online questionnaire link using the internal communication system, to invite qualified specialty nurses to participate in the survey. The principles of anonymity, confidentiality, and informed consent were explicitly stated on the first page of the questionnaire. To guarantee the completeness of the questionnaire and mitigate any potential bias from duplicate submissions, all questions were mandatory, and each IP address was restricted to submission only once. Following the survey, the researchers retrieved the data from the platform, excluding responses that were completed in 2 min or less, displayed consistent selections across all questions, and exhibited clear patterns. To ensure accuracy in data entry, a double-checking process was implemented. A total of 576 completed questionnaires were returned, of which 552 were deemed valid—a response rate of 95.83%. Among the 552 participants, there were 54 males and 498 females, aged 24–48 years (see Table 1). Of the questionnaires, 276 were designated as data for EFA, and 276 were used for the CFA. Additionally, to evaluate the test–retest reliability of the job stressor scale for specialty nurses, 30 participants were randomly selected and asked to complete a retest 2 weeks later.

**Table 1:** General information of the 552 specialty nurses.

Variables	Number	Percent (%)
Gender		
Male	54	9.78
Women	498	90.22
Age (years)		
≤30	96	17.39
31 ~ 35	261	47.28
36 ~ 40	159	28.80
41 ~ 45	30	5.43
≥46	6	1.09
Department		
Internal medicine	76	13.77
Surgery	92	16.67
Obstetrics	37	6.70
Pediatrics	32	5.80
Emergency	48	8.70
Severe	108	19.57
Operating room	72	13.04
Other	87	15.76
Education		
Junior college	24	4.35
Undergraduate	474	85.87
Master of Science	54	9.78
Title		
Nurse	78	14.13
Supervisor nurse	429	77.72
Deputy Director and above	45	8.15
Working years (years)		
1 ~ 10	180	32.61
11 ~ 20	348	63.04
≥21	24	4.35

### 2.5 Data analysis

SPSS (version 23.0) was used for the correlation analysis, EFA, and reliability evaluation, while AMOS 22.0 was used for the CFA. Categorical data were described using frequency and percentage, while continuous data that adhered to a normal distribution were presented as mean ± standard deviation. The reliability of the expert correspondence results was assessed using the positive coefficient of experts, authority coefficient, judgment basis coefficients, and Kendall’s coefficient of concordance (Wang, 2020). Item analysis was conducted using the critical ratio method (Mengmei et al., 2022). Pearson’s correlation coefficients, EFA, and CFA were employed to analyze the structural validity of the scale, including both intra-and interdimensional relationships with the total score (Li et al., 2023). Content validity was calculated based on expert ratings of the items (Zhao et al., 2022). Finally, the reliability of the scale was evaluated, using Cronbach’s alpha coefficients, test–retest reliability, and split-half reliability.

#### 2.5.1 Item analysis

The critical ratio method was used to sort the participants’ total scores, with the top 27% defined as the high-scoring group and the bottom 27% defined as the low-scoring group (Wu, 2010a,b). An independent *t*-test was conducted to compare the differences in scores for each item between the two groups. Items with a critical value of less than 3.000 or without statistically significant differences (*p* > 0.05) were identified (Zhang et al., 2021). The items were evaluated using criticality analysis, and the correlation coefficient with the total scale score was calculated. Items with low correlations were considered for removal.

#### 2.5.2 Content validity

Based on expert evaluations, the content validity index for the total scale (S-CVI) and the content validity index for each individual item (I-CVI) were rigorously calculated to ensure assessment precision and reliability. An I-CVI ≥ 0.780 and an S-CVI ≥ 0.900 indicates good content validity (Shi et al., 2012).

#### 2.5.3 Construct validity

An EFA, along with the correlation coefficient between each dimension and the correlation coefficient between a dimension and the total scale, was used to assess construct validity. The Kaiser–Meyer–Olkin (KMO) and the Bartlett’s test for sphericity values were calculated. A KMO value >0.700 and a significant Bartlett’s test (*p* < 0.05) indicated the suitability of the data for factor analysis. Common factors with eigenvalues ≥1.000 were extracted, and items with multiple loadings on factor loadings were removed. A scale structure was considered valid if the factor-loading value for each item exceeded 0.500, and the cumulative variance contribution was >50% (Yildiz et al., 2021). Additionally, a CFA was performed to validate the fit of the factor structure derived from the EFA with the new data.

#### 2.5.4 Reliability

A Cronbach’s *α* coefficient and split-half reliability were used to reflect the internal consistency reliability of the scale, as well as each facet. A Cronbach’s alpha coefficient greater than 0.700 and a split-half reliability coefficient exceeding 0.800 indicate excellent internal consistency (Sun and Xu, 2014; Wang et al., 2023). Furthermore, the stability of the scale was evaluated using the test–retest reliability coefficient, where a higher correlation coefficient between the results of the two surveys administered to the same group of subjects signified better scale stability (Yuan, 2021). Additionally, the greater the correlation between scores from consecutive surveys administered to the same cohort, the stronger the stability of the scale (Tsukuda et al., 2022).

## 3 Results

### 3.1 Preliminary item pool

During the literature review, a total of 4,120 relevant articles were identified, including 964 from CNKI, 434 from VIP, 360 from Wanfang, 1717 from PubMed, and 645 from Web of Science. After removing duplicate articles and sequentially reviewing titles, abstracts, and full texts, 18 articles were included for detailed analysis. We also drew inspiration from the NSS, pioneered by Gray-Toft and Anderson (1981), as a globally acclaimed assessment instrument for nursing stress. Li and Liu (2000) formulated the Chinese Nurse Stressor Scale based on the NSS, and through expert consultation; their scale exhibited good reliability and validity, albeit with limited specificity. Accordingly, the current study established a basic framework for the job stressor scale for specialty nurses, encompassing 29 items organized into five dimensions: specialty nursing and work (3 items), workload and time allocation (7 items), patient care (4 items), management and interpersonal relationships (9 items), and working environment and resources (6 items).

To ensure that the developed job stressor scale is more in accordance with the actual work environment and occupational characteristics of specialty nurses, we conducted structured interviews to supplement the item pool. Interview findings confirmed that the factors contributing to stress among specialty nurses align with the preliminary framework of the stressor scale. Moreover, issues such as low social status, unclear job responsibilities, frequent handling of patients with difficult or complex conditions, and undefined practice were also identified as significant stressors for specialty nurses. These findings enriched our research content and ensured that the construction of the specialty nurses’ job stressor scale items adopted a comprehensive and informed approach. Ultimately, the scale comprised 35 items across five dimensions: specialty nursing and work (5 items), workload and time allocation (9 items), patient care (6 items), management and interpersonal relationships (9 items), and working environment and resources (6 items).

### 3.2 Analysis of the Delphi survey results

Fourteen experts participated in this study after two rounds of correspondence, including eight undergraduates, five persons with a master’s degree, and one doctorate holder. Regarding professional titles, the experts included two with senior titles, eight with deputy senior titles, and four with intermediate titles. In terms of professional backgrounds, they included those of eight clinical nurses, three nursing managers, two nursing educators, and one psychological counselor. The average age was 41.57 ± 5.49 years, and they had an average of 20.36 ± 7.20 working years.

In the Delphi study, the positive coefficient of experts in the two rounds was 93.33 and 100%, the expert authority coefficient of the two rounds of expert consultations were 0.90 and 0.91, and the judgment basis coefficients of the experts in the two rounds were 0.95 and 0.96. Kendall’s *W* coefficients were 0.213 and 0.214 (*p* < 0.001), respectively, indicating good consistency in the consultation results. The importance scores ranged from 3.07 to 4.93 points, and the coefficients of variation were between 0.054 and 0.486.

Finally, the research team collated and discussed the opinions of experts. Six items were deleted, as their importance scores were <3.5 points. A further two items that did not align closely with the characteristics of specialty nurses were deleted after the group discussion; this means that a total of eight items were deleted. Three items with imprecise expressions were revised, based on expert suggestions. In the second round of expert consultation, the importance scores of the items ranged from 3.57 to 5 points, with a coefficient of variation of 0.090 to 0.324. Considering the experts’ opinions, the research team adjusted the expression “not having enough resources to meet the needs of specialized nursing work” to “not having enough resources (such as instruments, equipment, and consumables) to meet the needs of specialized nursing work.” After two rounds of expert consultation, 27 items were retained.

### 3.3 Item analysis

The results of the critical ratio test showed that the differences between the high- and low-scoring groups for each item on the scale were significant (*t* = 6.513–68.679, *p* < 0.001). The results of the correlation coefficient test showed that the correlation coefficients between the scale items and the total score ranged from 0.444 to 0.820 (all >0.4). Additionally, the results of the homogeneity test showed that the Cronbach’s *α* coefficients did not increase after item deletion, indicating good consistency of the scale. None of the items were deleted using these three analytical methods.

### 3.4 Validation results

#### 3.4.1 Content validity

The individual content validity index (I-CVI) at the entry level ranged between 0.790 and 1.000, while the scale content validity index (S-CVI) was at 0.980. These findings suggest that the Specialty Nurses’ Work Stressor Scale has excellent content validity.

#### 3.4.2 Construct validity

An EFA was performed using the data obtained from the 552 specialty nurses, which initially generated four factors. The *χ*^2^ value of the Bartlett’s test of sphericity, measuring the suitability of the data for factor analysis, was 6312.863 (*p* = 0.916), indicating its adequacy for analysis. The subsequent principal component analysis, employing maximum variance orthogonal rotation, revealed a high correlation between the scores of “management and interpersonal relations” and “work environment and resources.” Consequently, these two dimensions were consolidated into a single dimension, labeled “work resources and environment.” The subsequent EFA revealed eigenvalues greater than one for the four factors, accounting for a cumulative variance contribution rate of 69.10%. A screen plot analysis further suggested a transition point for the four factors, indicating the appropriateness of retaining them. The factor-loading matrix revealed factor loadings exceeding 0.5, with individual item loadings ranging from 0.575 to 0.841 for their respective factors, indicating no evidence of multiple loadings. Table 2 provides a detailed overview of these findings. The correlation coefficients among the various factors ranged from 0.465 to 0.705, while the correlation coefficients between each factor and the overall scale ranged from 0.706 to 0.932 (see Table 3).

**Table 2:** Factor load matrix after rotation of the work stressor scale for specialty nurses (*n* = 276).

Items	Factor 1	Factor 2	Factor 3	Factor 4
Specialized Nursing and Work				
1. The social status of specialty nurses is not high	**0.644**	0.128	0.104	0.415
2. Nursing specialist work is difficult, heavy responsibility	**0.602**	0.337	0.240	0.302
3. The scope of practice of specialty nurses is not clear	**0.832**	0.117	0.174	0.290
4. The responsibilities of specialty nurses are not clear	**0.841**	0.081	0.183	0.349
5. Less autonomy in specialized nursing work	**0.707**	0.058	0.216	0.400
Workload and Time Allocation				
6. Too much specialized knowledge to learn	−0.010	**0.673**	0.222	0.190
7. We need to constantly update the frontier knowledge of specialty	−0.138	**0.757**	0.169	0.027
8. There are too many teaching tasks related to specialized nursing	0.239	**0.755**	0.116	0.239
9. There are too many research tasks related to specialized nursing	0.281	**0.730**	0.077	0.247
10. Medical institutions have too high requirements for the comprehensive ability assessment of specialty nurses	0.327	**0.706**	0.184	0.149
Patient Care				
11. Specialist care is not recognized by patients and their families	0.467	0.209	**0.575**	0.222
12. The patient ‘s condition is difficult or complex	0.246	0.391	**0.657**	0.109
13. Unforeseen changes in the condition of patients in care	0.190	0.281	**0.719**	0.094
14. Specialized knowledge cannot meet the complex psychological needs of patients and their families	0.272	0.194	**0.705**	0.311
15. Concern that specialist capacity does not meet patients ‘needs for specialist care	0.094	0.071	**0.806**	0.416
16. Concerns about the inability of specialist capacity to perform nursing consultations for complex and difficult patients	0.029	0.071	**0.797**	0.364
Work Resources and Environment				
17. Inadequate resources (e.g., instruments, equipment, and consumables) to meet the needs of specialist care	0.063	0.107	0.361	**0.640**
18. The physical and mental health of specialty nurses has received little attention	0.313	0.220	0.233	**0.677**
19. The professional development path of specialty nurses is vague	0.404	0.152	0.220	**0.735**
20. There are few opportunities for specialty nurses to continue their studies	0.277	−0.031	0.196	**0.798**
21. Opportunities for promotion of specialty nurses are limited	0.267	0.100	0.172	**0.801**
22. The distribution of performance bonuses does not match specialist care	0.342	0.274	0.058	**0.759**
23. Nursing managers have high expectations for specialty nurses	0.164	0.339	0.228	**0.629**
24. Nursing managers do not have enough understanding and support for specialty nurses	0.211	0.171	0.191	**0.792**
25. Lack of understanding and cooperation from colleagues in specialized nursing work	0.264	0.158	0.188	**0.754**
26. The new technology and new business of specialized nursing have not been recognized and supported by doctors	0.333	0.354	0.152	**0.648**
27. The level of support provided by family members for specialized nursing work is inadequate	0.179	0.154	0.281	**0.680**

Factor 1: specialized nursing and work, Factor 2: workload and time allocation, Factor 3: patient care, and Factor 4: work resources and environment. The bold values indicate that the factor loadings are >0.500, and can be classified as one type of dimension in a rotated factor-loading matrix.

**Table 3:** The correlation between each factor and the total scale.

Items	Specialized nursing and work (Factor 1)	Workload and time allocation (Factor 2)	Patient care (Factor 3)	Work resources and environment (Factor 4)	Total scale
Specialized nursing and work (Factor 1)	1.000				
Workload and time allocation (Factor 2)	0.465**	1.000			
Patient Care (Factor 3)	0.577**	0.544**	1.000		
Work resources and environment (Factor 4)	0.705**	0.534**	0.640**	1.000	
Total scale	0.823**	0.706**	0.810**	0.932**	1.000

**p < 0.05.

**Figure 1:**
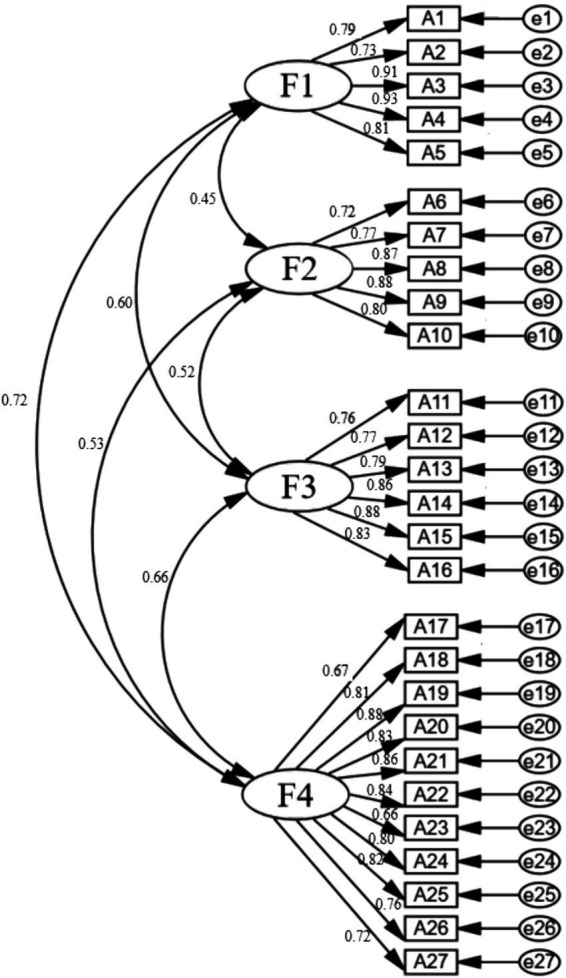
Structural validity of confirmatory factor analysis. A1–A27 represent scale items, while e1–e27 represent residuals.

The extracted factor structure was evaluated using a CFA and data were obtained from 276 of the participants. The goodness-of-fit of the four-factor structural model was tested. During the evaluation process, the goodness-of-fit of the scale’s dimensions and entries was assessed using the maximum likelihood method. The results demonstrated that the scale possessed favorable model fit indices, including *χ*^2^/df = 2.240, CFI = 0.931, IFI = 0.947, NFI = 0.909, and CFI = 0.947, along with RMSEA = 0.067 and RMR = 0.044. These indices confirm that the model fit was satisfactory, thereby verifying the scale’s robust construct validity. Detailed results are presented in Figure 1.

### 3.5 Reliability analysis results

As shown in Table 4, the overall Cronbach’s alpha coefficient (*α*) for the scale was 0.958, indicating excellent internal consistency. The Cronbach’s *α* coefficients of each dimension were 0.905, 0.841, 0.896, and 0.950 (see Table 4). Furthermore, the overall split-half reliability of the scale was 0.868, and the split-half reliability of each dimension were 0.900, 0.815, 0.813, and 0.912, respectively. Additionally, the overall test–retest reliability of the scale was 0.946, and the test–retest reliability of each dimension were 0.881, 0.893, 0.860, and 0.894.

**Table 4:** Cronbach’s alpha coefficient for specialty nurse job stressor scale.

Dimensions	Cronbach’s alpha coefficient	Eigenvalues	Cumulative contribution rate (%)
Specialized nursing and work	0.905	4.152	41.356
Workload and time allocation	0.841	3.590	69.096
Patient care	0.896	3.900	55.801
Work resources and environment	0.950	7.014	25.978

## 4 Discussion

Using a standardized and rigorous questionnaire development process, this study formulated the Specialty Nurses’ Work Stressor Scale, specifically for specialty nurses. During questionnaire development, the scale was tailored to the actual work situation and psychological state of specialty nurses in China, enabling a more objective assessment of their current job stress levels. Our findings confirm that the scale demonstrated good internal consistency and validity. With acceptable explanatory variance, it accurately and effectively evaluated the stressors and stress levels faced by specialty nurses in their work. Having a scale to measure these factors allows for the development of targeted intervention strategies to reduce occupational burnout caused by work stress, encourage specialty nurses to actively engage in their work, and significantly enhance the quality of specialized nursing services in medical institutions (Qin et al., 2022a,b).

This is the first study to focus exclusively on specialty nurses and aim to develop a detailed scale to assess their work stressors and stress levels. Compared with previous scales designed for a wide range of nurses, this study’s goal was to be more specialized and develop a measure that is tailored specifically for the specialty nurse population. The Specialty Nurse Work Stressor Scale comprises four dimensions and a total of 27 items. The scale comprehensively covers four crucial domains: “specialized nursing and work,” “workload and time allocation,” “patient care,” and “work resources and environment.” A five-point Likert scale was employed, with each item scored on a spectrum ranging from 1 = “I do not experience this feeling” to 5 = “I experience this feeling very strongly.” This enabled specialty nurses to objectively describe the job stressors they encounter daily. Moreover, the scale contains a moderate number of items, takes approximately 3–5 min to complete, and has clear and easily understandable content. This makes it convenient for specialty nurses to complete, highly doable, and practical.

Considering the specificity and complexity of specialty nurses’ work, this study-based on the framework of the Robbins’ work stress model-drew on the most widely used Chinese Nurse Work Stressor Scale, after thorough literature research. By integrating the professional characteristics of Chinese specialty nurses with the outcomes of structured interviews, we constructed a comprehensive item pool for the specialty nurse work stressors scale. To ensure the rigor and scientificity of the scale, 14 authoritative experts from diverse units and related fields participated in a two-round Delphi consultation process. The overall Cronbach’s *α* coefficient, split-half reliability coefficient, and test–retest reliability all demonstrated the scale’s high internal consistency and stability.

Furthermore, the analysis of the KMO index indicated that the data were suitable for factor analysis. Exploratory factor analysis identified four major factors, which were inconsistent with the structures of other scales (Li and Liu, 2000; Sarıalioğlu et al., 2022). In the original design of the scale, “management and interpersonal relationships” and “work resources and environment” were two separate dimensions. However, the scores of the specialty nurse participants on these two aspects were highly correlated, suggesting that they belonged to the same factor. This convergence can be attributed to the interdependency of work resources, which encompasses both the intrinsic and extrinsic factors that a job offers employees. Intrinsic resources, such as compensation for labor and professional growth opportunities, are inherently linked to the job itself, while extrinsic resources pertain to interpersonal relationships, including support and recognition from leaders and colleagues (Walsh et al., 2015). Consequently, after thorough deliberation among the research team, the factors were consolidated into a single dimension, titled “Work resources and environmental aspects.” After revision, the fitting indices of the confirmatory factor analysis met the statistical requirements. The resulting job stressor scale for specialty nurses comprises 4 dimensions and 27 items, demonstrating good reliability and validity, and is capable of assessing the sources and degree of stress among specialty nurses.

Unlike other stress measurement tools (Gray-Toft and Anderson, 1981; Cohen et al., 1983; Lee and Cha, 2021), our scale is based on the Robbins stress model, focusing on the sources of work stress rather than merely the superficial reactions to it. This approach significantly enhances the depth and accuracy of stress assessments, enabling more precise identification and location of stress factors in the workplace and providing clear directions for designing and implementing targeted interventions. Moreover, this study considered the distinctive characteristics of specialty nurses, including their specialized work content, significant workload, special working environment, diverse service recipients, rapid evolution of professional knowledge and skills, and high societal expectations (Qin et al., 2022a,b). This scale can be used to comprehensively evaluate the sources and extent of work-related stress among specialty nurses. The scale could also enhance specialty nurses’ awareness of their own stress state, helping them to adopt positive coping strategies such as seeking support, adjusting work habits, or engaging in relaxation training. Additionally, the scale provides nursing managers with a foundation to enhance the utilization and management strategies of specialty nurses, thereby mitigating occupational health risks, promoting their physical and mental well-being, enhancing motivation, and ultimately facilitating the high-quality development of the specialty nursing profession.

## 5 Limitations

Despite its clear contributions, this study had some limitations. First, the degree of criterion correlation, which is an important indicator for assessing the validity of the scale, was not evaluated. Second, the sample was confined to specialty nurses from two tertiary general hospitals in Zhengzhou City, Henan Province, which limits the representativeness of the results. Furthermore, the scale was developed based on specialty nurses in China, and as such, certain questions or expressions may hold different connotations or levels of acceptance among specialty nurse groups in different countries, potentially limiting the cross-cultural applicability of the scale. To address these limitations, future research should consider a wider range of participants to validate and refine the scale, thereby enhancing its generalizability and applicability.

## 6 Conclusion

The job stressor scale for specialty nurses developed in this study comprises 27 items across four dimensions: specialty nursing and work, workload and time allocation, patient care, working environment and resources. The scale exhibits good reliability and validity, with clear and comprehensible content and strong operability. It can effectively serve as a valuable tool for assessing job stressors among specialty nurses and provide insights into the specific challenges they face in their professional environments.

## Data Availability

The raw data supporting the conclusions of this article will be made available by the authors, without undue reservation.
